# Case report: Adult case of A20 haploinsufficiency suspected as neuro-Behçet disease

**DOI:** 10.3389/fimmu.2024.1508307

**Published:** 2025-01-07

**Authors:** Harumi Shirai, Naoko Saito-Sato, Emiko Horiuchi, Hirotoshi Kikuchi, Saori Kadowaki, Hidenori Ohnishi, Takeshi Suzuki

**Affiliations:** ^1^ Department of Rheumatology & Allergology, Japanese Red Cross Medical Center, Tokyo, Japan; ^2^ Department of Neurology, Japanese Red Cross Medical Center, Tokyo, Japan; ^3^ Institute of Medical Genomics, International University of Health and Welfare, Chiba, Japan; ^4^ General Medical Education and Research Center, Teikyo University, Tokyo, Japan; ^5^ Department of Early Diagnosis and Preventive Medicine for Rare Intractable Pediatric Diseases, Graduate School of Medicine, Gifu University, Gifu, Japan; ^6^ Department of Pediatrics, Graduate School of Medicine, Gifu University, Gifu, Japan

**Keywords:** neuro-Behçet disease, A20 haploinsufficiency, diplopia, TNFAIP3, variant functional analysis

## Abstract

Patients with A20 haploinsufficiency (HA20) presenting with central nervous system (CNS) symptoms are rare, and available reports are limited. Here, we describe a patient with HA20, previously followed up as Behçet disease, who presented with CNS symptoms in adulthood. A 38-year-old Japanese male who had been followed up for incomplete Behçet disease at another hospital since 28 years of age presented to our hospital with acute-onset diplopia and persistent hiccups that were severe enough to cause vomiting. Despite suspicion of neuro-Behçet disease on the basis of the patient’s medical history, a definitive diagnosis could not be made. He experienced transient episodes of diplopia over a short period, and brain magnetic resonance imaging T2 fluid-attenuated inversion recovery images revealed nonspecific hyperintensities in the cerebral white matter. He was initially managed with low-dose prednisolone and colchicine but continued to experience low-grade fever, recurrent oral ulcers, and genital ulcers. A gene panel test for periodic fever syndromes revealed a variant in the *TNFAIP3* gene, showing a c.259C>T nonsense variant. As previous reports have described the same variant in patients with HA20, the patient was diagnosed with HA20. The patient’s response to glucocorticoids and colchicine therapy was limited, and his symptoms improved upon initiation of tumor necrosis factor-α inhibitor therapy. The variant showing a c.259C>T nonsense variant in the *TNFAIP3* gene has been previously reported in China and France, making this the first report in Japan, which is considered a rare instance of HA20 with CNS involvement.

## 1 Introduction

In 2016, Zhou et al. reported a novel autoinflammatory disorder presenting with Behçet disease-like symptoms at a young age that was caused by embryonic heterozygous variants in the *TNFAIP3* gene and named as “A20 haploinsufficiency” (HA20) (1). Expression of the A20 protein encoded by the mutated *TNFAIP3* gene is reduced by half, leading to dysregulation of inflammatory cytokines, such as tumor necrosis factor-alpha (TNF-α), interleukin-6 (IL-6), and interleukin-1 (IL-1) (2). In patients with HA20, the overproduction of such inflammatory cytokines is believed to cause Behçet disease-like symptoms, e.g., recurrent oral ulcers, fever, arthralgia, and gastrointestinal ulcers, at a young age. Additionally, there have been reports of concurrent autoimmune diseases, such as Hashimoto thyroiditis, systemic lupus erythematosus (SLE), and autoimmune hepatitis, as well as various other symptoms, including vitiligo, lymphoproliferative changes, and uveitis. Persistent inflammation leads to progressive damage of various organs. To date, approximately 200 cases have been reported, with most patients diagnosed during childhood. Some patients have been diagnosed in adulthood in families with affected children (3, 4). Patients presenting with central nervous system (CNS) symptoms are even rarer, and available reports are limited. Here, we describe a patient with HA20, previously followed up as Behçet disease, who presented with CNS symptoms in adulthood.

## 2 Case description

A 38-year-old Japanese male presented to our emergency department with a chief complaint of unknown onset diplopia and persistent hiccups. He had a medical history of fever, oral ulcers, arthralgia, epididymitis, genital ulcers, and folliculitis at 28 years of age and was previously diagnosed with incomplete Behçet disease at another hospital. He had been self-administering colchicine and low-dose prednisolone (PSL) at a dosage of 0–5 mg/day for oral and genital ulcers. No substantial brainstem lesions were observed on magnetic resonance imaging (MRI), and the patient was managed conservatively. However, after 6 months, he experienced recurrent diplopia lasting for approximately 4 days, along with headaches and low-grade fever; therefore, he self-increased his dosage of PSL to 6–10 mg/day. Although his symptoms improved, there was concern that he might have neuro-Behçet disease and was admitted to our hospital for further evaluation and treatment.

Upon admission, his vital signs were within normal limits, and the physical examination revealed preexisting oral ulcers but no new skin lesions, including erythema nodosum. The neurological examination findings were unremarkable, with no evidence of diplopia. Laboratory test results showed a white blood cell count of 7030/μl, hemoglobin level of 14.7 g/dl, platelet count of 33.5 × 10^4^/μl, and a C-reactive protein level of 1.67 mg/dl, with no evidence of liver or kidney dysfunction. Coagulation test and urinalysis findings were unremarkable. Although the anti-nuclear antibody was weakly positive at a titer of 80× for a speckled pattern, other autoimmune antibodies including the anti-dsDNA, anti-Sm, anti-CLβ2GPI, anti-cardiolipin, anti-aquaporin 4, anti-MOG, and anti-SS-A antibodies were all negative, not meeting the classification criteria for SLE or other autoimmune diseases. Despite a previous diagnosis of incomplete Behçet disease at 28 years of age, the patient did not harbor the HLA-B51 allele, and the pathergy test results were negative. He had a history of suspected atypical mycobacterial infection at 36 years of age; however, results of the Capilia MAC antibody ELISA test and sputum culture were negative. He had no family history of autoimmune or autoinflammatory diseases, including Behçet disease.

**Figure 1:**
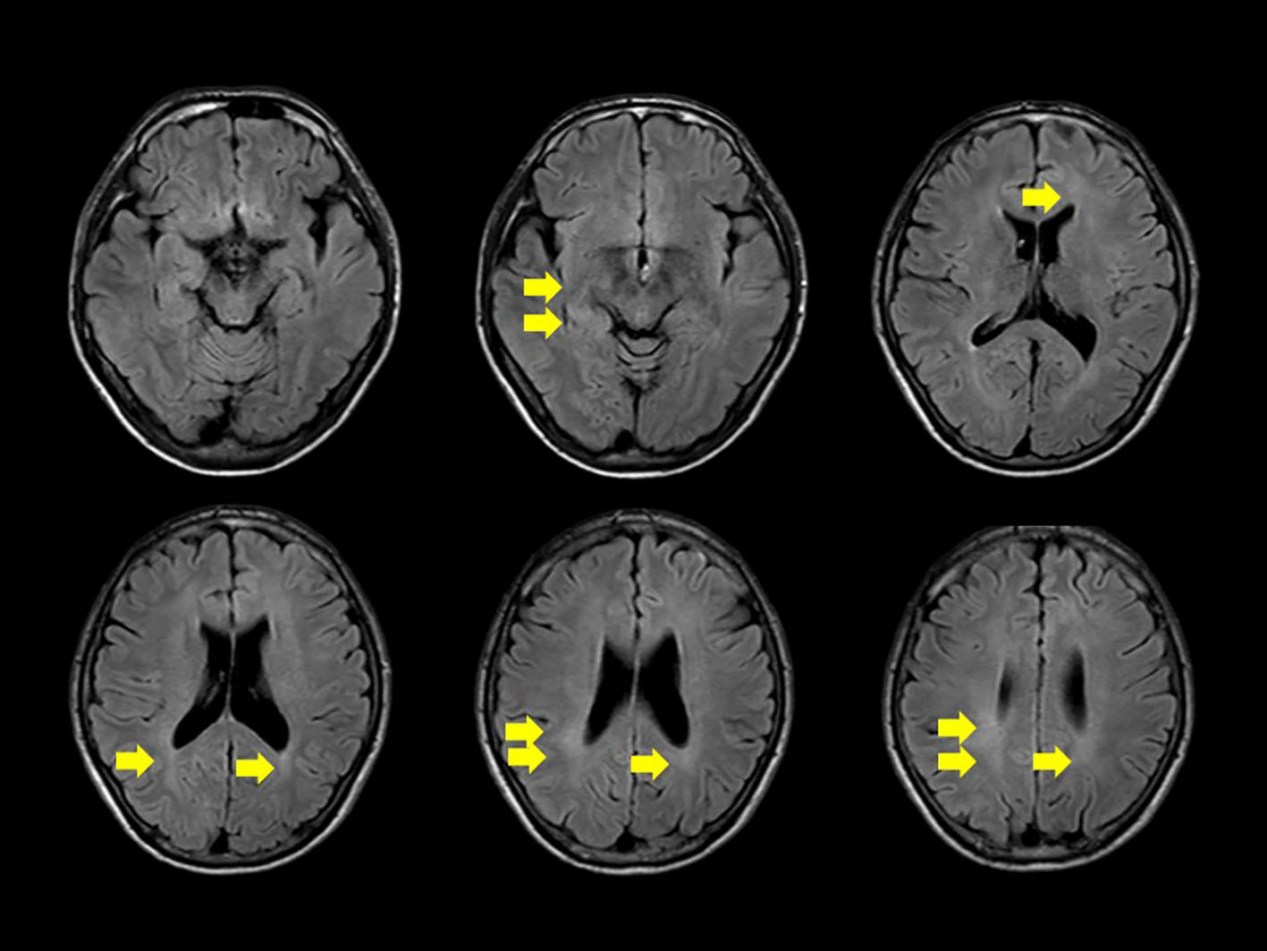
Diffuse, faint high signal on fluid-attenuated inversion recovery is observed in the deep white matter on head magnetic resonance imaging (yellow arrow). No apparent signs of meningitis, tumorous lesions, abnormal signals or enhancement, pathological ventricular enlargement, or brainstem atrophy are noted.

We conducted a comprehensive investigation to determine the cause of visual impairment and elevated inflammatory markers. Brain MRI revealed diffuse faint fluid-attenuated inversion recovery (FLAIR) hyperintensity in the deep white matter. No obvious tumor lesions, pathological enlargement of the ventricles, abnormal signals, or brainstem atrophy were observed. Diffusion MRI showed no infarction, and magnetic resonance angiography did not reveal any considerable stenosis or aneurysms (**Figure 1**). MRI of the cervical, thoracic, and lumbar spines revealed no substantial abnormalities. According to the cerebrospinal fluid (CSF) analysis, the CSF IL-6 level was 17.8 pg/ml (serum IL-6, 2.6 pg/ml), CSF sIL-2R levels were below commercially available detection sensitivity (<100 U/ml), and CSF cell count was 2/μl (lymphocytes, 2; polymorphonuclear leukocytes, <1). The CSF cultures, including those for acid-fast bacilli and general bacteria, tested negative. Self-adjustment of PSL was not performed, and the patient continued PSL at a dosage of 3.5 mg/day. According to the international consensus recommendation criteria for diagnosing neuro-Behçet disease (5), definite neuro-Behçet disease must meet all of the following criteria: 1) satisfy the International study Group Criteria 1990 or any other accepted current or future criteria for Behçet disease; 2) recognize neurological syndrome (with objective neurological signs) to be caused by Behçet disease and supported by relevant and characteristic abnormalities seen on neuroimaging and/or CSF analysis; and 3) lack of a better explanation for the neurological findings. As a reference finding, it has also been stated that cerebrospinal fluid tests show inflammatory changes such as increases in cells, protein concentrations, and IL-6 levels. In our case, CSF analysis was repeated after a 3-week interval, showing a decrease in the CSF IL-6 level to 6.7 pg/ml. In our patient, CSF and MRI imaging data were not consistent with typical manifestations of neuro-Behçet disease.

Chest computed tomography (CT) revealed centrilobular nodular opacities in both lung fields, with bronchiectasis suggestive of nontuberculous mycobacterial infection and chronic respiratory tract infection, which improved over the past year with no new shadows or pleural effusion. Abdominal contrast CT did not reveal any obvious findings indicative of a source of fever or inflammation. General bacterial and acid-fast bacillus cultures from the sputum samples were negative. Upper and lower gastrointestinal endoscopies revealed only gastric fundic gland polyps, with no other abnormalities, such as gastrointestinal ulcers. The ophthalmological examination did not reveal any substantial ocular abnormalities indicative of uveitis or visual dysfunction causing diplopia.

After further examination and outpatient observation, bilateral tinnitus and decreased hearing in the right ear were observed. The patient was diagnosed with acute sensorineural hearing loss and started PSL at a dosage of 20 mg, which resulted in an improvement in hearing. The PSL dosage was tapered to 5 mg. Subsequently, we observed an improvement in neurological symptoms with a slight increase in the PSL dosage, and we noted episodes of fever or low-grade fever that occurred approximately once a month. Considering the patient’s history of recurrent oral ulcers since 10 years of age, we suspected hereditary periodic fever syndrome. Gene panel testing related to hereditary autoinflammatory diseases (K010-14_v13, Kazusa DNA Research Institute) was conducted and targeted *ADA2, NLRC4, TNFAIP3, MEFV, TNFRSF1A, NRLP3, NLRP12, MVK, PLCG2*, and *NOD2*. The results revealed a heterozygous variant of the *TNFAIP3* gene, specifically c.259C>T (R87X) (**Table 1**). This variant has been previously reported in two families with HA20 (6, 7).

**Table 1:** Variants found as a result of genetic testing associated with autoinflammatory disease.

No.	Gene name	Feature ID	Genotype	HGVS.c	HGVS.p	ToMMo 54KJPN AF*	gnomAD Global AF**
1	*TNFAIP3*	NM_006290.4	Heterozygous	c.259C>T	p.Arg87Ter	No registration	6.195e-7
2	*NLRP3*	NM_004895.4	Heterozygous	c.214G>A	p.Val72Met	1.011e-2	4.400e-4
3	*PLCG2*	NM_002661.5	Heterozygous	c.1959C>T	p.Arg653=	3.266e-2	3.940e-4

Next-generation sequencing was used to analyze the genetic base sequences of *ADA2*, *NLRC4*, *TNFAIP3*, *MEFV*, *TNFRSF1A*, *NRLP3*, *NLRP12*, *MVK*, *PLCG2*, and *NOD2* genes associated with hereditary autoinflammatory diseases, and variant detection was performed. *Tohoku Medical Megabank Organization (ToMMo) 54KJPN allele frequency (AF): 54KJPN is a SNV/INDEL allele/genotype frequency panel constructed from short-read whole genome sequencing analyses of approximately 54 000 Japanese individuals (11). These data were obtained from https://jmorp.megabank.tohoku.ac.jp/. **gnomAD version 4.1.0 Global AF: The Genome Aggregation Database (gnomAD) version 4.1.0 has been previously described (12). These data were searched for at https://gnomad.broadinstitute.org/. No., number.

**Figure 2:**
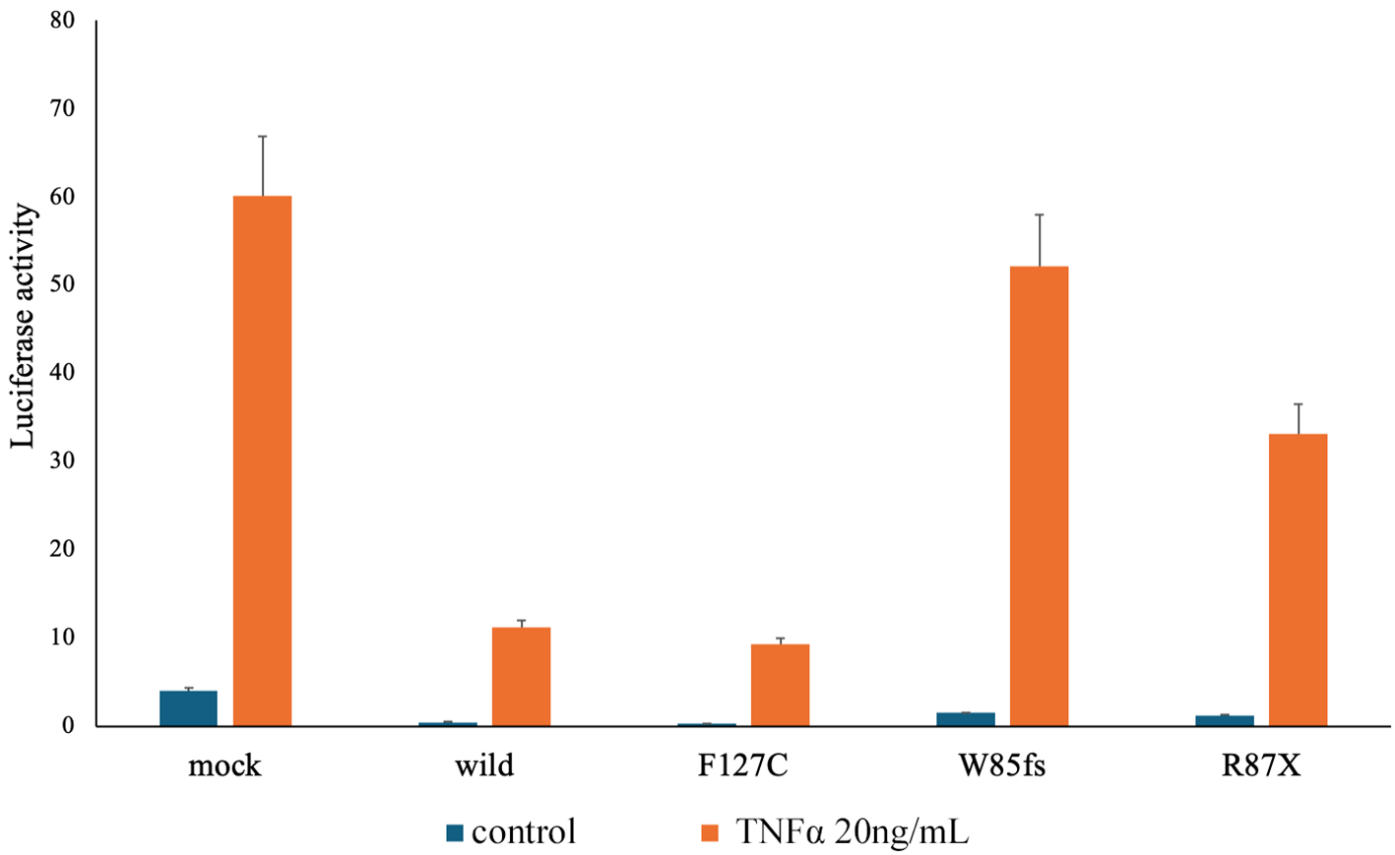
Result of the *TNFAIP3* gene variant function analysis. To confirm whether the patient had a disease-related variant in the *TNFAIP3* gene, we conducted functional analysis of variants of the same gene using a previously reported method (8). The Dual-Luciferase Reporter Assay System (Promega) was used to perform a reporter gene assay that examines nuclear factor kappa B (NF-κB) expression in response to tumor necrosis factor stimulation. For the variant observed in this case as R87X on the right end, compared with the wild type, the Luciferase activity is higher, indicating a substantial failure of NF-κB activity inhibition, confirming it as a pathological variant. Mock: A20 haploinsufficiency (A20)-deficient human embryonic kidney (HEK) 293 cells were transfected with 10 ng of pcDNA3.1+ mock vector per well. Wild-type: A20-deficient HEK293 cells were transfected with 10 ng of pcDNA3.1+ myc-A20 wild-type per well. F127C: *TNFAIP3* gene single nucleotide polymorphisms as the negative variant control. W85fs: *TNFAIP3* gene frameshift variant as the pathogenic variant control. R87X: *TNFAIP3* gene c.159C>T variant (present case).

To confirm whether the *TNFAIP3* variant was disease-related, we performed variant functional analysis of the gene using a previously reported method (8) (**Figure 2**). Wild-type and mutant plasmid vectors of myc-tagged *TNFAIP3* were transfected into A20-deficient human embryonic kidney (HEK) 293 cells, along with a pcDNA3.1+ myc-empty vector as the control. Additionally, the cells were co-transfected with a plasmid for the nuclear factor kappa B (NF-κB) reporter gene assay. After 24 hours of incubation, cells were cultured for 6 hours either without stimulation or with TNF-α stimulation at 20 ng/ml, followed by cell recovery. Subsequently, a luciferase reporter activity assay was performed to detect luminescence. The R87X variant identified in this case exhibited considerably higher luciferase activity than the wild-type, indicating a pathological variant with impaired inhibition of NF-κB activity. On the basis of these findings, the patient was diagnosed with HA20. The variants identified in the *NLRP3* and *PLCG2* genes, as shown in **Table 1**, were assessed to have limited disease relevance. Monogenic Behçet disease-like symptoms caused by variants in the *RELA* gene have also been reported (9, 10). We further confirmed the *RELA* gene but found no variants in this gene. We re-evaluated the patient’s family history and symptoms through inquiries.

**Figure 3:**
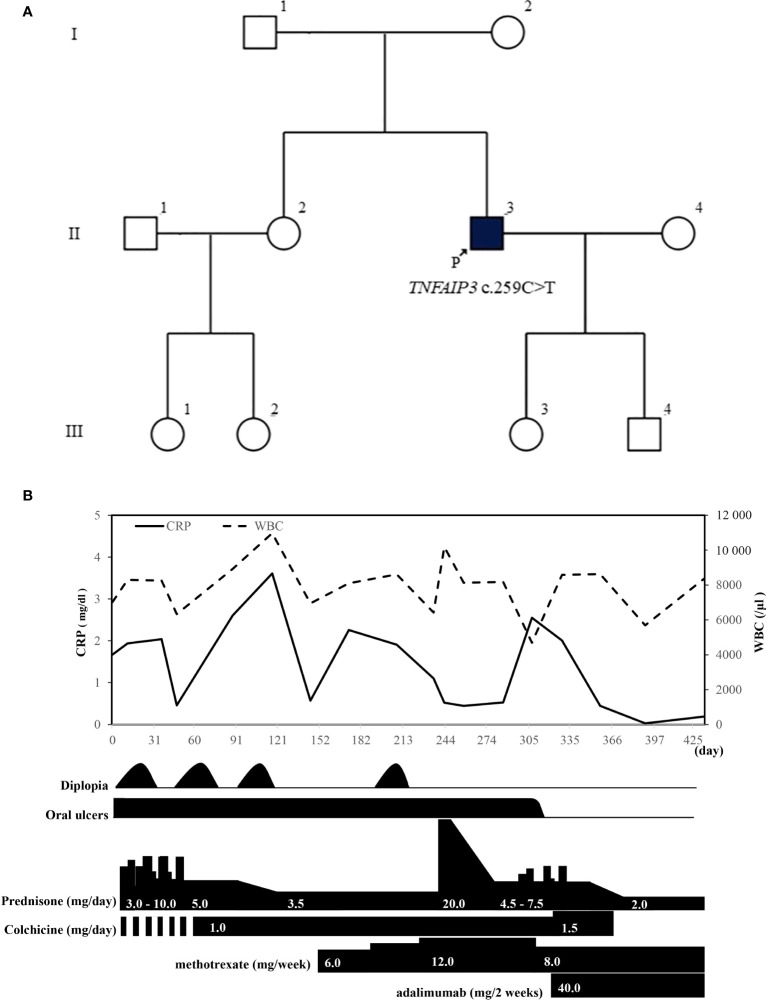
**(A)** In addition to the proband (II-3), his elder sister (II-2), niece (III-1), and daughter (III-3) exhibit symptoms of oral ulcers suggestive of A20 haploinsufficiency. **(B)** Patient’s clinical course after visiting our hospital. WBC, white blood cell; CRP, C-reactive protein.

His relatives, namely his elder sister (II-2), niece (III-1), and daughter (III-3), besides the index case (II-3), exhibited symptoms suggestive of HA20 with oral ulceration (**Figure 3A**). Genetic testing has not been conducted on any of his relatives at present. Initially suspected of neuro-Behçet disease, the patient received an increased dosage of PSL and additional treatment with methotrexate (12 mg/week), but he showed a poor therapeutic response. After the definitive diagnosis, bronchoscopy was performed owing to a history suggestive of non-tuberculous mycobacterial infection, and tissue cultures were obtained, confirming the absence of notable pathogens. After explaining the possibility of a certain risk of infection and obtaining the patient’s consent, we started administration of adalimumab. After starting adalimumab, the oral ulcers disappeared, inflammatory signs became negative for the first time, and there were no episodes of diplopia thereafter. Colchicine was discontinued owing to diarrhea, and tapering of prednisolone to a dosage of 2 mg/day became possible (**Figure 3B**).

## 3 Discussion

In this report, we described a case in which neurological symptoms emerged during follow-up for Behçet disease, leading to a diagnosis of HA20. Regarding HA20, the variant of the *TNFAIP3* gene, c.259C>T (R87X), observed in this case, has been confirmed in two families from China and France (6, 7). This variant has not been registered in the Tohoku Medical Megabank Organization, a Japanese genome database (11) and is an extremely rare variant according to information registered in the Genome Aggregation Database (12) (**Table 1**). Functional analysis of the *TNFAIP3* gene c.259C>T (R87X) variant has not been previously reported, and in this study, we performed that for the first time, confirming that the variant is pathogenic by showing a substantial impairment in regulation of NF-κB activity compared with the wild type.

In this case, neuro-Behçet disease was initially suspected, and an elevation of the IL-6 level was confirmed in cerebrospinal CSF analysis. However, little is known about the normal expected levels of IL-6 in the CSF of patients with neuro-Behçet disease. Hirohata et al. previously reported that CSF IL-6 was a useful biomarker for the diagnosis of chronic progressive neuro-Behçet disease at the cut-off value of 16.55 pg/ml (13). Additionally, they reported the neuro-Behçet disease algorithm (14), but the patient CSF IL-6 level of 17.8 pg/ml was borderline. Adeeb et al. reported that *RELA* loss-of-function mutations cause Behçet disease-like mucocutaneous ulcerative syndrome and neuromyelitis optica via impaired NF-κB signaling (10). Considering the possibility of a variant in the *RELA* gene, we investigated variants in the *RELA* gene but found no variants.

CNS symptoms in HA20 have been reported worldwide, albeit infrequently (4, 7, 15). In a study by Kadowaki et al. that summarized HA20 cases in East Asia, CNS symptoms were reported in 4.2% (3 of 71) of the patients, suggesting that CNS symptoms are rare in HA20 cases (3). Reports of patients initially diagnosed as having Behçet disease, leading to the diagnosis of HA20, have been sporadic. Typically, lesions in neuro-Behçet disease tend to occur in the brainstem, diencephalon, and basal ganglia, whereas CNS lesions in HA20 are reported to be distributed throughout the cerebral cortex to cerebral vessels. Various brain histology such as necrotizing granulomatous inflammation, small vessel vasculitis, and central venous thrombosis have been reported (4, 16, 17). Among cases of CNS involvement in HA20, the CSF cell count varied by case, with some cases falling within normal limits and others showing a considerable increase in the cell count associated with aseptic meningitis (4, 18, 19). Elhani et al. conducted a systematic literature review of studies on patients with *TNFAIP3* sequence variants. They reported that 17 of 177 (9.6%) patients displayed a wide spectrum of CNS manifestations, including abnormal brain parenchymal lesions as well as unexplained developmental disorders, epilepsy, and cerebral palsy due to neonatal cardiac arrest (20). **Table 2** shows characteristics of only HA20 patients with abnormal lesions in the brain parenchyma or abnormal CSF findings. Only cases 11 and 12 were from the same family; all others were from independent families. In cases other than case 12, it took approximately 10–20 years for CNS symptoms to appear.

**Table 2:** Comparison of our case with other published cases of A20 haploinsufficiency characterized by CNS involvement.

No.	Age at onset/Current age	Sex/Ethnicity	TNFAIP3 HGVS.c, HGVS.p	Domain location	Affected brain location and major CNS symptoms	Current treatment	Reference
1	18 years/ 38 years	M/Japanese	c.259C>T, p.Arg87*	OTU	Diplopia, MRI diffuse FLAIR hyperintensity in the deep white matter	GC, MTX, & ADA	Our case
2	10 years/25 years	F/European American	c.671delT, p.Phe224Serfs*4	OTU	SLE with CNS vasculitis (based on brain imaging findings), chorea, & migraine	Anakinra, AZA, & GC→Tofacitinib	(1, 4, 15)
3	2 years/ 20 years	F/European Canadian	c.680T>A, p.Leu277*	OTU	Headache as a CNS symptom	Infliximab	(1)
4	4 years/17 years	F/Dutch	c.918C>G, p.Tyr306*	OTU	Cerebral palsy due to perinatal lacunar infarction	Infliximab	(4)
5	1 week/8 years	M/unknown (adopted)	c.1012G>T, p.Glu338*	OTU	Right arytenoid mass, inflammatory fibroepithelial polyp, frontal lobe punctate, & small vessel CNS vasculitis	Died	(4)
6	6 months/6 years	F/Indian	c.1504C>T, p.Arg502Trp	2^nd^ ZnF	Headache, type 2 Arnold Chiari malformation	Colchicine & AZA	(25)
7	10 years/29 years	F/Chinese	c.1690A>T, p.Lys564*	Between the 3^rd^ & 4^th^ ZnF	Cerebral venous thrombosis, cerebral infarction, & hemiplegia	GC & CPA	(17)
8	2.5 years/14 years	F/American	C.1777C>T, p.Gln593*	Between the 3^rd^ & 4^th^ ZnF	Headache, aseptic meningitis (7.5 years)	GC, MMF, RTX, HCQ, & IVIg	(19)
9	32 years/ 36 years	F/Chinese	c.1806delG, p.T602fs*95	4^th^ ZnF	NPSLE involving both the central and peripheral nervous system (bilateral ptosis, grand mal seizure, & difficulty looking upward)	GC, MMF, &CPA	(26)
10	6 months/8 years	F/Pakistani-Indian	c.1939A>C, p.Thr647Pro	4^th^ ZnF	Left-sided focal seizures, hemiparesis, & intracranial mass lesions in the gray matter of the paracentral lobule & the thalamus on the left side. Brain histology revealed necrotizing granulomatous inflammation	GC & baricitinib	(16)
11	Childhood/33 years	M/Japanese	c.2209delC, p.Gln737Serfs*79	6^th^ ZnF	Craniopharyngioma	Levothyroxine for Hashimoto’s thyroiditis	(18, 27)
12	2 months/11 months	F/Japanese	c.2209delC, p.Gln737Serfs*79	6^th^ ZnF	Aseptic meningitis	Colchicine	(18)

ADA, adalimumab; AZA, azathioprine; CNS, central nervous system; CPA, cyclophosphamide; F, female; GC, glucocorticoid; HCQ, hydroxychloroquine; IVIg, intravenous immunoglobulin; M, male; MMF, mycophenolate mofetil; MTX, methotrexate; NPSLE, neuropsychiatric systemic lupus erythematosus; OTU, ovarian tumor domain; RTX, rituximab; SLE, systemic lupus erythematosus; ZnF, Zinc finger domain.

The genotype of all cases was heterozygous. Cases 11 and 12 are from the same family lineage.

HA20 is a monogenic autoinflammatory disease with highly variable clinical manifestations. Clinical heterogeneity of HA20 has been previously described, even among the same relatives (4). In HA20 patients with the same variant (c.259C>T, R87X) as in our case, typical symptoms of oral ulcers along with arthralgia, interstitial lung disease, and liver dysfunction have been reported; however, CNS symptoms were not observed (6, 7). The *TNFAIP3* gene is located on the chromosome 6q23.3 and contains an amino-terminal ovarian tumor (OTU) domain followed by seven zinc finger (ZnF) domains. Chen et al. reported differences in phenotype depending on the variant domain that exists (21). They reported that the onset of HA20 is earlier in patients with ZnF domain disruption than in those with OTU domain disruption. Additionally, they reported that disruption of the ZnF domain, compared with the OTU domain, might be more closely related to musculoskeletal disorders. However, they did not mention CNS involvement. Regarding the domain location of the variants with CNS symptoms, there were five cases at the OTU domain and seven cases at the ZnF domain. Because the reported number of HA20 patients with CNS symptom is small, it is difficult to determine a clear trend. Heterogeneity of HA20 also might indicate the possibility of additional effects with other modifying alleles and genetic, epigenetic, and/or environmental risk factors, such as vaccinations or infections, and/or aging. Further elucidation of the etiology and phenotype-genotype correlation analysis are needed.

Although a diffuse, faint FLAIR hyperintensity was observed in the deep white matter in our patient, no specific findings directly attributable to diplopia were identified on imaging. It is worth considering that the evaluation was not conducted using thin MRI slices, which may have limited the depiction of the lesions. To date, there have been no reports of HA20 cases that specifically mention deep white matter lesions or symptoms of diplopia. The possibility that the sensorineural hearing loss observed during the patient’s clinical course was also a symptom of HA20 was considered. In animal models, chronic neurological inflammation persisted in A20-deficient mice, and the inflammation worsened with age (22). In the present case, the IL-6 concentration in the CSF may have been persistently high. Because the long-term effects of adalimumab on CNS symptoms have not been established, follow-up of clinical symptoms and CSF laboratory findings is important.

The European League Against Rheumatism recommend that acute attacks in neuro-Behçet disease with parenchymal involvement should be treated with high-dose glucocorticoids followed by slow tapering, together with immunosuppressives, such as azathioprine. However, cyclosporine should be avoided. Monoclonal anti-TNF antibodies should be considered in severe disease as first-line treatment or in refractory patients (23). Yet, there is no standard treatment for HA20, and current treatment recommendations are symptom oriented. There are reports of treatment with glucocorticoids, non-steroidal anti-inflammatory drugs, colchicine, thalidomide, and both conventional and biologic disease-modifying antirheumatic drugs, including methotrexate, azathioprine, sulfasalazine, cyclosporine, TNF-α inhibitors, tocilizumab, and anakinra. Additionally, treatment with hematopoietic stem cell transplantation has been reported (15, 20). Because patients with HA20 show overproduction of TNF and IL-1β, biologics targeting these cytokines are often used in severe cases. Moreover, the presence of a type I IFN signature predicted a good response to JAK inhibitors in HA20 patients with CNS involvement who were resistant to biologic agents (4, 15, 16).

HA20 typically manifests in early childhood, at approximately 5 years of age. In contrast to Behçet disease, the genetic pattern remains unclear, although HA20 demonstrates autosomal dominant inheritance. Variations such as recurrent fever, ulcers that exhibit scar healing, and the detection of autoantibodies have been reported in HA20 (24). While many reports of HA20 originate from pediatric cases, other conditions such as undifferentiated fever or Behçet disease may be suspected, emphasizing the importance of detailed history-taking to confirm the presence of childhood symptoms, such as oral ulcers, genital ulcers, and family history. In patients with a history of oral ulcer episodes since a young age and prolonged inflammatory findings, HA20 should be considered in the differential diagnosis. Furthermore, the fundamental genetic pattern of HA20 is autosomal dominant inheritance, necessitating not only treatment strategies for the affected individual, but also proactive genetic counseling to predict the risk of onset in the next generation.

Here, we described a rare case of HA20 with CNS involvement. The long-term prognosis of HA20, including CNS involvement, remains uncertain, and the establishment of effective treatment strategies is desired through further accumulation of cases.

## Data Availability

The original contributions presented in the study are included in the article/supplementary material. Further inquiries can be directed to the corresponding author.
